# Spatiotemporal trends in hospital morbidity indicators due to road traffic injuries in the state of Piauí, Brazil, 2010–2023

**DOI:** 10.1590/1980-549720260044

**Published:** 2026-08-24

**Authors:** Francisco Gabriel Thomaz Bastos, Gabriel Stumpf Bastos Amorim, Francisco Antônio da Cruz Dos Santos, Malvina Thaís Pacheco Rodrigues, Márcio Dênis Medeiros Mascarenhas

**Affiliations:** I Universidade Federal do Piauí – Teresina (PI), Brazil.; II Universidade Federal do Piauí, Center for Intelligence on Emerging and Neglected Tropical Diseases – Teresina (PI), Brazil.

**Keywords:** Traffic accident, Hospitalization, Hospital Information Systems, Mortality, Time Series Studies

## Abstract

**Objective:**

To analyze spatiotemporal trends in hospital morbidity indicators for road traffic injuries in the state of Piauí, Brazil, from 2010 to 2023.

**Methods:**

This is an ecological, time-series and spatial analysis using data from the Hospital Information System of the Brazilian Unified Health System. Trends in hospitalization rates, according to health regions and sociodemographic characteristics, were analyzed using annual percent change (APC) and 95% confidence intervals (95%CI), estimated by Prais-Winsten regression. Spatial clusters were identified at the municipality level using Local Indicators of Spatial Association (LISA) maps.

**Results:**

A total of 80,251 hospitalizations due to road traffic injuries were recorded. The mean hospitalization rate was 176.1/100 thousand inhabitants, showing an increasing trend (APC 5.72; 95%CI 1.04; 10.61), while case fatality (2.3%) remained stable (APC -2.42; 95%CI -5.07; 0.30). The mean hospitalization cost per admission (BRL 1,123.92) showed a decreasing trend (APC -3.17; 95%CI -5.57; -0.71), whereas the mean length of hospital stay (4.9 days) remained stable (APC -1.07; 95%CI -2.96; 0.86). High-high spatial clusters of hospitalization rates were observed in municipalities located in the Northern and Southern regions of the state.

**Conclusion:**

Hospitalization rates for road traffic injuries increased in Piauí, followed by a reduction in mean cost and stability in case fatality and length of hospital stay. Higher morbidity was concentrated in the Northern and Southern regions, suggesting a geographic shift of hospital morbidity toward inland areas.

## INTRODUCTION

Road Traffic Injuries (RTI) represent an important global public health issue. According to the Global Burden of Disease (GBD) 2023, road transport injuries remain among the main causes of disability-adjusted life years (DALYs) worldwide, with a nonsignificant reduction of 13.3% in age-standardized rates between 2010–2023^1^. Data from GBD 2021 show that, in 2021, there were approximately 103.2 million cases of road transport injuries globally, with higher mortality rates in low- and middle-income countries (22.6 per 100 thousand inhabitants), when compared to high-income countries². Men aged 15 to 49 years represent more than 60% of years lived with disability globally^2^.

In Brazil, RTI have significant magnitude. The prevalence of self-reported injuries in 12 months was 2.4% in 2019, with significant regional disparities^3^. Mortality reached 21.0 deaths per 100 thousand inhabitants in 2013, with over one million potential years of life lost that year, especially in the age group of 20–29 years^4^. Estimates adjusted for 2019 suggested a 25% decline in deaths since 2012, although official statistics underestimated the actual number of deaths by 31%^5^. Motorcyclists represent the most affected group: deaths increased from 4.1% of the total in 1996 to 28.5% in 2013, with an 820% increase in mortality rate^4^. In the city of São Paulo, hospitalization rates among motorcyclists increased again from 2015 onward, exceeding 140 per 100 thousand inhabitants among men aged 20 to 59 years in 2019^6^.

Among the implemented public policies, the New Drinking and Driving Law [*Lei Seca*] (Law 12.760/2012), which established zero tolerance for alcohol and more severe punishments, showed a significant impact on reducing mortality and hospitalizations due to RTI^5^. The total economic cost of RTI in urban regions was estimated at approximately USD 9.9 billion in 2006, equivalent to 1.2% of the Brazilian GDP of that year^4^.

In the state of Piauí, RTI consist in a great health and social challenge. The state recorded one of the highest growth rates in the risk of dying from RTI in Brazil among non-capital cities, with a mean increase of 7.50% per year in the period from 2000 to 2016^5^.

The analysis of hospital morbidity according to health regions and sociodemographic characteristics is paramount to support regional public policies of prevention and control. Knowledge of spatiotemporal patterns of hospitalization can guide the allocation of resources, the planning of emergency and trauma services, and the implementation of preventive interventions directed to populations and regions of higher risk. Furthermore, the analysis of hospital cost trends allows us to evaluate the economic impact on the state health system and support decisions on investments in prevention compared to treatment costs. In the present study, we aimed to analyze spatiotemporal trends of indicators of hospital morbidity due to road traffic injuries in the state of Piauí, Brazil, from 2010 to 2023.

## METHODS

### Study design and period

This is an ecological, time-series study with spatial analysis, conducted with data on hospitalizations due to RTI involving residents of Piauí from 2010 to 2023. The time frame was defined considering that 2010 marks the beginning of the Decade of Action for Road Safety, proposed by the United Nations (UN), and that 2023 corresponded to the last year with consolidated data in the information system used at the time of the study.

### Study population

The study population comprised all hospital admissions due to RTI (ICD-10: V01-V89) recorded in the Hospital Information System of the Brazilian Unified Health System (*Sistema de Informações Hospitalares* – SIH/SUS), of residents of the state of Piauí, from 2010 to 2023.

### Study location

The study location is the state of Piauí, in the Northeast of Brazil. It has 224 municipalities, 12 health regions, and four health macroregions. The population consisted of 3,271,199 inhabitants in 2022, with a territorial area of 251,755.48 km^2^ and population density of 12.99 inhabitants/km^2^. Its Human Development Index (HDI) was 0.690 in the year 2021, ranking 23rd among the 27 Federative Units (FU)^7^. The state has the second highest proportion of motorcycles per household in Brazil. According to the Continuous National Household Sample Survey of the Brazilian Institute of Geography and Statistics (IBGE), about 597 thousand households had motorcycles in 2024, corresponding to 52.1% of the state's households^7^.

### Data source

Data on hospital morbidity were obtained from SIH/SUS, being processed as aggregated data via TABNET. The SIH/SUS is a nationwide information system, fed by Hospital Admissions Authorizations (*Autorizações de Internação Hospitalar* – AIH), which are filled by health facilities that provide hospital care to the Brazilian Unified Health System (SUS). AIH contains information on diagnoses, performed procedures, patient's demographics, length of stay, hospitalization outcome, and paid amounts. After completion, AIH is processed by municipal and state managers and consolidated by the SUS Department of Informatics (*Departamento de Informática do SUS* – DATASUS). SIH/SUS covers approximately 75% of hospital admissions in Brazil, with regional variations^8^.

Population data were collected from the study *Projeção da População das Unidades da Federação por sexo e grupos de idade:* *2000–2030* ("Projection of the Federative Units’ population by sex and age groups: 2000–2030"), developed by the Ministry of Health.

All data used in this study are available on the DATASUS website (https://datasus.saude.gov.br/informacoes-de-saude-tabnet/), which were collected in August 2024.

### Study variables

Hospitalizations due to RTI were identified by the secondary diagnosis recorded in the AIH, which corresponds to the external cause of hospitalization, according to Chapter XX of the International Statistical Classification of Diseases and Related Health Problems, 10th Revision (ICD-10). The secondary diagnosis, in this context, refers to the circumstance of the accident that motivated the injury recorded as the main diagnosis. The considered categories were: pedestrian (V01-V09), cyclist (V10-V19), motorcyclist (V20-V39), car, truck, and bus occupants (V40-V79), other types of transport or unspecified condition of the victim (V80-V89), and total accidents of road transport (V01-V89).

The records were stratified according to: age group—children (0 to 9 years), adolescents (10 to 19 years), young adults (20 to 39 years), adults (40 to 59 years), and older adults (60 years or over); sex—men and women; type of victim—pedestrian, cyclist, motorcyclist, vehicle occupant, others; residence health macroregion and its respective health regions—Semi-arid (composed of the Vale do Canindé, Vale do Rio Guaribas, Serra da Capivara, and Chapada das Mangabeiras health regions), Mid North (Cocais, Entre Rios, Vale do Sambito, and Carnaubais health regions), Coastline (Planície Litorânea and Chapada Vale do Rio Itaim health regions), and Cerrado (Tabuleiros do Alto Parnaíba and Vale dos Rios Piauí e Itaueiras health regions). The mean cost of hospitalization was also collected as a variable.

### Indicators of hospital morbidity

The following indicators were calculated for the state of Piauí and for each level of disaggregation (sex, age group, type of victim, health region, and health macroregion):

- Hospitalization rate per 100 thousand inhabitants: ratio between the number of hospitalizations due to RTI and the resident population corresponding to the analyzed stratum (total population for Piauí; male or female population for the sex stratum; population of the respective age group for the age group stratum; and resident population in the respective health region or macroregion for the geographical strata), multiplied by 100,000;
- Hospital case fatality per 100: ratio between the number of hospitalizations due to RTI whose outcome was death in the hospital and the number of hospitalizations due to RTI multiplied by 100;
- Mean cost of hospitalization: total amount paid for hospitalizations due to RTI divided by the number of hospitalizations due to RTI;
- Mean length of hospital stay (days): total days of hospitalization due to RTI divided by the number of hospitalizations due to RTI.

Data referring to the total hospitalization cost in Brazilian currency (reais, BRL) were submitted to monetary correction by applying the General Price Index – Market (*Índice Geral de Preços –Mercado*)^9^, using the calculator of the Central Bank of Brazil^10^.

### Statistical analysis

Hospital morbidity indicators (dependent variable: Y) were transformed into logarithmic scale (log_10_) to stabilize the variance and allow the interpretation of the coefficients as percent change. The temporal trend analysis was performed using the generalized linear regression of Prais-Winsten, which corrects the serial autocorrelation of the residuals through an iterative transformation based on the estimation of the first-order autocorrelation coefficient (ρ). The presence of serial autocorrelation was evaluated by the Durbin-Watson statistic. Based on the β coefficients and standard errors estimated by the model, the Annual Percent Change (APC) and the respective 95% confidence intervals (95%CI) were calculated, according to the formula^11^: APC=(10^β^−1)×100. The applied hypothesis test was the t-test for the β regression coefficient, with a significance level of p<0.05. Trends were classified as increasing (p<0.05 and positive β), decreasing (p<0.05 and negative β), or stationary (p≥0.05), according to criteria previously described in the literature^12^.

Spatial analysis was conducted in three time intervals (2010–2014, 2015–2019, and 2020–2023), established to capture possible changes in spatial patterns over the period. Spatial analysis was restricted to the hospitalization rate because it is the indicator that best reflects the geographic distribution of the morbidity burden. Initially, choropleth maps were drawn with the accumulated hospitalization rates of the respective periods. Subsequently, the Local Indicator of Spatial Association (LISA) was used to identify spatial clusters, classifying the municipalities according to their association patterns as high-high, low-low, high-low, and low-high^13^. Only clusters with statistical significance (p<0.05) were considered, the others being classified as nonsignificant. The neighborhood matrix was defined by the "Queen" method.

Data were tabulated in Microsoft Excel and converted into indicators. Trend analysis was performed in Stata, version 16.1 (StataCorp LP, College Station, USA). Spatial analyses were carried out using the Python software, version 3.10.3, with the ESDA library^14^, version 2.7.0, and official digital maps of the IBGE.

### Ethical aspects

This study exclusively used secondary, anonymized, and public-access data, without any identification of the participants. Therefore, it is exempted from consideration by the Research Ethics Committee, according to the Resolutions of the National Health Council No. 466/2012 and No. 510/2016, which provide for the waiver of ethical evaluation for research with public data.

#### Data Availability Statement:

The entire dataset that supports the results of this study is available upon request to the corresponding author. The dataset is not publicly available due to the integration of databases created by the authors.

## RESULTS

**Figure 1:**
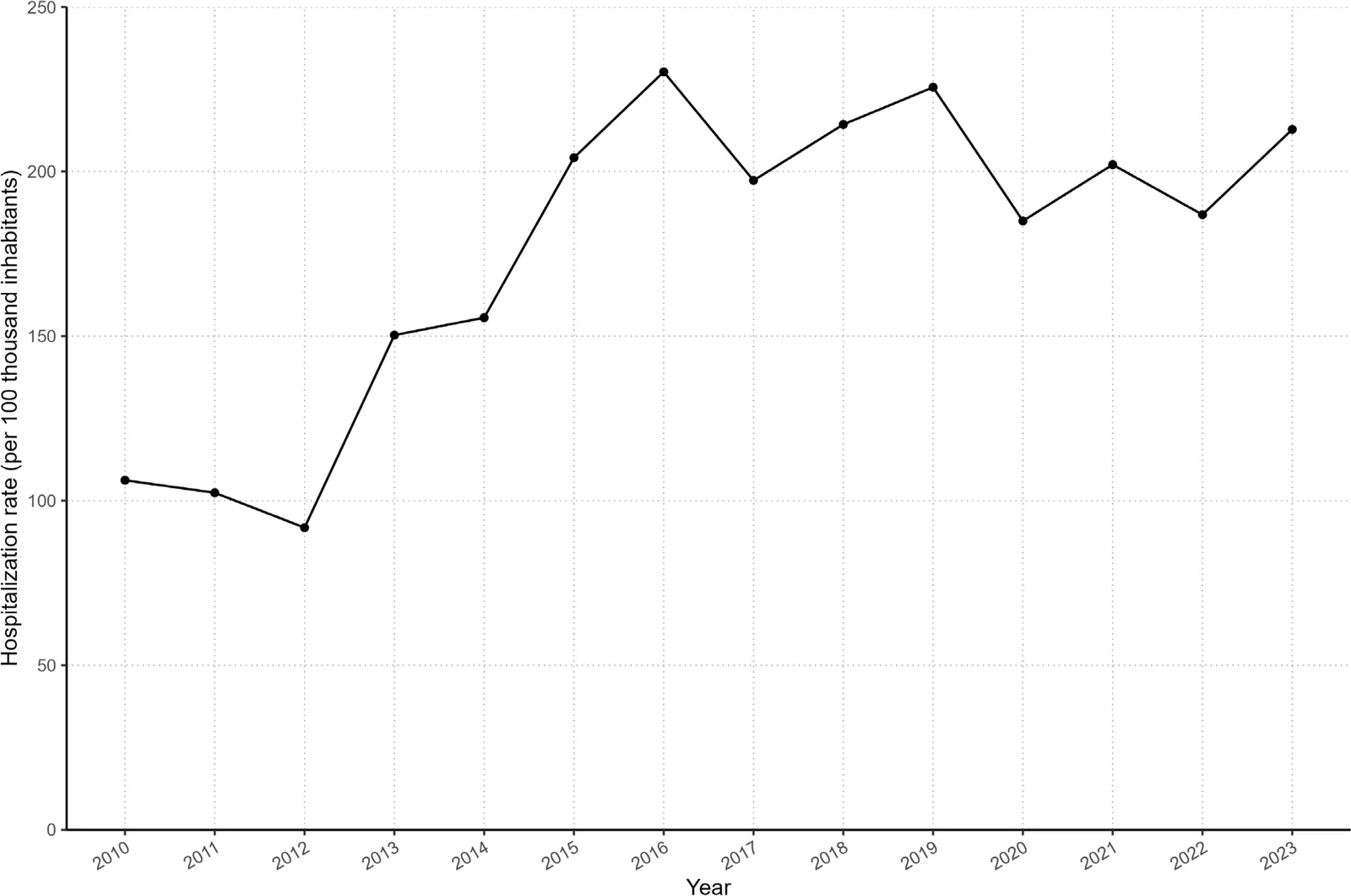
Time series of the rate of hospitalization due to road traffic injuries per 100 thousand inhabitants, Piauí, Brazil, 2010–2023 (n=80,251).

Source: Hospital Information System of the Brazilian Unified Health System (SIH/SUS/DATASUS) and population projections of the Ministry of Health; prepared by the authors.

Between 2010 and 2023, 80,251 hospitalizations due to RTI were recorded in Piauí, with a mean rate of 176.1 hospitalizations/100 thousand inhabitants and an increasing trend (APC 5.72; 95%CI 1.04 to 10.61). The year with the greatest number of hospitalizations was 2016 (n=7,478; 230.3/100 thousand inhab.) and the smallest was 2012 (n=2,950; 91.8/100 thousand inhab.), as demonstrated in Table 1 and Figure 1.

**Table 1:** Rate of hospitalization due to road traffic injuries per 100 thousand inhabitants and temporal trend, Piauí, 2010–2023 (n=80,251).

Variables		Hospitalization rate	APC (95%CI)	p-value	Trend
Piauí		176.1	5.72 (1.04; 10.61)	0.020	Increasing
Sex					
	Men	292.6	5.71 (0.92; 10.71)	0.023	Increasing
	Women	65.9	6.13 (1.77; 10.69)	0.009	Increasing
Age (in years)					
	0–9	24.5	5.20 (-2.66; 13.70)	0.180	Stationary
	10–19	124.8	4.56 (-0.79; 10.19)	0.089	Stationary
	20–39	279.1	4.30 (0.03; 8.74)	0.048	Increasing
	40–59	200.6	7.54 (2.75; 12.55)	0.005	Increasing
	60 or over	112.8	8.47 (2.40; 14.90)	0.010	Increasing
Type of victim					
	Pedestrian	12.6	-0.60 (-11.50; 11.64)	0.912	Stationary
	Cyclist	4.3	13.73 (2.05; 26.74)	0.024	Increasing
	Motorcyclist	151.2	5.76 (1.32; 10.39)	0.015	Increasing
	Vehicle occupant	4.7	11.07 (-0.64; 24.16)	0.063	Stationary
	Others	3.3	6.60 (-3.53; 17.8)	0.188	Stationary
Health region					
	Carnaubais	272.6	12.92 (1.05; 26.19)	0.035	Increasing
	Chapada das Mangabeiras	171	3.50 (-2.96; 10.39)	0.267	Stationary
	Cocais	110.1	3.41 (-2.94; 10.17)	0.271	Stationary
	Entre Rios	230.7	8.52 (2.13; 15.31)	0.012	Increasing
	Planície Litorânea	84.4	-1.34 (-24.14; 28.32)	0.913	Stationary
	Serra da Capivara	181.9	7.44 (5.65; 9.26)	<0.001	Increasing
	Tabuleiros do Alto Parnaíba	243.1	10.63 (4.04; 17.62)	0.004	Increasing
	Vale do Canindé	171.2	-2.64 (-6.62; 1.52)	0.189	Stationary
	Vale do Rio Guaribas	83.8	-13.84 (-19.29; -8.03)	<0.001	Decreasing
	Vale do Sambito	122	-1.29 (-10.03; 8.29)	0.765	Stationary
	Vale dos Rios Piauí e Itaueiras	227.2	7.17 (4.24; 10.17)	0.001	Increasing
	Chapada Vale do Rio Itaim	54.2	-11.38 (-20.04; -1.78)	0.025	Decreasing
Macroregion					
	Semi-arid	100.1	-7.20 (-11.62; -2.56)	0.006	Decreasing
	Mid North	235.6	9.03 (2.05; 16.49)	0.015	Increasing
	Coastline	99.5	3.92 (-4.66; 13.27)	0.350	Stationary
	Cerrado	199.5	6.23 (4.72; 7.76)	<0.001	Increasing

**APC:** Annual Percent Change; 95%CI: 95% confidence interval. For age, sex, health region, and macroregion, rates were calculated using the population corresponding to each stratum analyzed as denominator.

**Source:** Hospital Information System of the Brazilian Unified Health System (SIH/SUS/DATASUS) and population projections of the Ministry of Health; prepared by the authors.

Hospitalization rate among men (292,6/100 thousand men) was 4.4 times higher than that of women (65.9/100 thousand women), with an increasing trend in both sexes. For age group, young adults (20–39 years) presented the highest mean rate (279.1/100 thousand inhab.) of age group, followed by adults aged 40–59 years (200.6/100 thousand inhab.). Both groups, as well as those of older adults, showed an increasing trend, while children and adolescents presented stationary values (Table 1).

Motorcyclists concentrated the highest burden of hospitalizations, with a mean rate of 151.2/100 thousand inhabitants—12 times higher than that of pedestrians (12.6/100 thousand inhab.). Only motorcyclists and cyclists showed increasing trend. Among the health regions, Carnaubais recorded the highest rate (272.6/100 thousand inhab.) of the region, and Chapada Vale do Rio Itaim, the lowest (54.2/100 thousand inhab.). A decreasing trend was observed in Vale do Rio Guaribas, Chapada Vale do Rio Itaim, and in the Semi-arid macroregion; the others showed increasing or stationary trends (Table 1).

In the period, there were 1,866 hospital deaths, with a mean case fatality of 2.3% and a stationary trend. A decreasing trend of case fatality was observed only in the Entre Rios region, in the Mid North macroregion, and in the age groups of young adults and older adults. The other geographic strata showed a stationary trend (Table 2).

**Table 2:** Hospital case fatality due to road traffic injuries and temporal trend, Piauí, 2010–2023 (n=80,251).

Variables		Hospital case fatality (%)	APC (95%CI)	p-value	Trend
Piauí		2.3	-2.42 (-5.07; 0.30)	0.076	Stationary
Sex					
	Men	2.5	-2.38 (-5.15; 0.48)	0.094	Stationary
	Women	1.6	-2.11 (-4.60; 0.44)	0.096	Stationary e
Age (in years)					
	0–9	1.4	-0.65 (-7.76; 7.02)	0.853	Stationary
	10–19	1.7	-0.93 (-5.52; 3.88)	0.675	Stationary
	20–39	2.1	-2.99 (-5.58; -0.33)	0.031	Decreasing
	40–59	2.3	-2.55 (-2.55; 0.60)	0.103	Stationary
	60 or over	5.1	-5.36 (-8.14; -2.50)	0.002	Decreasing
Type of victim					
	Pedestrian	2.4	4.33 (-5.96; 15.75)	0.391	Stationary
	Cyclist	2.4	1.82 (-8.25; 12.99)	0.713	Stationary
	Motorcyclist	2.3	-2.93 (-6.05; 0.29)	0.071	Stationary
	Vehicle occupant	3.6	4.40 (-5.89; 15.82)	0.384	Stationary
	Others	2.1	-5.69 (-16.93; 7.06)	0.334	Stationary
Health region					
	Carnaubais	2.1	-5.95 (-12.19; 0.72)	0.075	Stationary
	Chapada das Mangabeiras	1.7	1.41 (-6.73; 10.25)	0.722	Stationary
	Cocais	3.6	1.07 (-2.82; 5.12)	0.566	Stationary
	Entre Rios	2.1	-5.34 (-8.75; -1.81)	0.007	Decreasing
	Planície Litorânea	1.3	-10.5 (-20.98; 1.38)	0.076	Stationary
	Serra da Capivara	2.5	10.42 (0.02; 21.91)	0.050	Stationary
	Tabuleiros do Alto Parnaíba	1.7	-1.49 (-8.19; 5.70)	0.651	Stationary
	Vale do Canindé	1.9	1.25 (-7.87; 11.28)	0.779	Stationary
	Vale do Rio Guaribas	5.1	9.04 (-3.19; 22.81)	0.139	Stationary
	Vale do Sambito	3.6	-1.05 (-6.43; 4.65)	0.689	Stationary
	Vale dos Rios Piauí e Itaueiras	1.8	0.86 (-3.94; 5.91)	0.709	Stationary
	Chapada Vale do Rio Itaim	4.8	-1.35 (-15.08; 14.59)	0.846	Stationary
Macroregion					
	Semi-arid	3.7	2.86 (-7.84; 14.80)	0.586	Stationary
	Mid North	2.1	-5.21 (-8.92; -1.36)	0.013	Decreasing
	Coastline	2.8	-0.03 (-4.05; 4.16)	0.988	Stationary
	Cerrado	1.9	3.17 (-0.53; 7.01)	0.087	Stationary

**APC:** Annual Percent Change; 95%CI: 95% confidence interval.

**Source:** Hospital Information System of the Brazilian Unified Health System (SIH/SUS/DATASUS); prepared by the authors.

The total cost of hospitalizations was BRL 90.2 million (BRL 128.0 million in values corrected by the IGP-M). The mean cost per hospitalization was BRL 1,123.92 (corrected: BRL 1,659.40), with decreasing trend (APC -3.17; 95%CI -5.57 to -0.71). No stratum showed an increasing trend for this indicator (Table 3).

**Table 3:** Mean value of hospital admissions due to road traffic injuries and temporal trend, Piauí, 2010–2023 (n=80,251).

Variables		Mean cost (BRL)	APC (95%CI)	p-value	Trend
Piauí		1,123.92	-3.17 (-5.57; -0.71)	0.016	Decreasing
Sex					
	Men	1,151.85	-3.00 (-5.43; -0.52)	0.022	Decreasing
	Women	1,006.70	-3.90 (-6.20; -1.54)	0.004	Decreasing
Age (in years)					
	0–9	958.08	-3.44 (-6.47; -0.31)	0.034	Decreasing
	10–19	1,110.28	-4.31 (-6.80; -1.76)	0.003	Decreasing
	20–39	1,126.98	-2.82 (-5.28; -0.29)	0.032	Decreasing
	40–59	1,118.48	-3.23 (-5.38; -1.03)	0.008	Decreasing
	60 or over	1,187.77	-3.22 (-6.09; -0.26)	0.035	Decreasing
Type of victim					
	Pedestrian	974.28	-4.53 (-14.18; 6.21)	0.362	Stationary
	Cyclist	984.04	0.80 (-1.02; 2.64)	0.360	Stationary
	Motorcyclist	1,132.64	-2.96 (-5.14; -0.73)	0.014	Decreasing
	Vehicle occupant	1,258.30	2.51 (-1.50; 6.68)	0.201	Stationary
	Others	1,283.86	-8.28 (-15.15; -0.85)	0.032	Decreasing
Macroregion					
	Semi-arid	1,191.10	-1.29 (-5.60; 3.21)	0.537	Stationary
	Mid North	1,155.13	-3.69 (-6.89; -0.38)	0.032	Decreasing
	Coastline	1,124.11	-7.03 (-11.80; -2.00)	0.011	Decreasing
	Cerrado	1,005.40	-2.15 (-3.66; -0.62)	0.010	Decreasing

**APC:** Annual Percent Change; 95%CI: 95% confidence interval.

**Source:** Hospital Information System of the Brazilian Unified Health System (SIH/SUS/DATASUS); prepared by the authors.

Hospitalizations totaled 392,047 days of length of stay, with a mean time of 4.9 days and a stationary trend. The mean length of hospital stay was predominantly stationary, with a decreasing trend only among children and adolescents and increasing among vehicle occupants (Table 4).

**Table 4:** Mean length of stay (days) of hospital admissions due to road traffic injuries and temporal trend, Piauí, 2010–2023 (n=80,251).

Variables		Mean length of stay (days)	APC (95%CI)	p-value	Trend
Piauí		4.9	-1.07 (-2.96; 0.86)	0.248	Stationary
Sex					
	Men	4.9	-1.00 (-2.92; 0.96)	0.287	Stationary
	Women	4.6	-1.38 (-3.21; 0.48)	0.130	Stationary
Age (in years)					
	0–9	3.6	-2.26 (-3.29; -1.22)	0.001	Decreasing
	10–19	4.7	-1.57 (-2.99; -0.13)	0.035	Decreasing
	20–39	4.8	-1.22 (-3.04; 0.62)	0.173	Stationary
	40–59	5.1	-1.36 (-3.50; 0.82)	0.198	Stationary
	60 or over	5.5	-1.12 (-3.19; 0.98)	0.266	Stationary
Type of victim					
	Pedestrian	5.4	-0.9 (-2.05; 0.27)	0.119	Stationary
	Cyclist	4.2	-1.05 (-3.96; 1.93)	0.453	Stationary
	Motorcyclist	4.8	-1.12 (-3.29; 1.10)	0.290	Stationary
	Vehicle occupant	5.7	5.84 (1.15; 10.74)	0.018	Increasing
	Others	6.3	-2.9 (-5.74; 0.02)	0.051	Stationary
Macroregion					
	Semi-arid	5.3	1.42 (0.06; 2.81)	0.042	Increasing
	Mid North	4.2	-2.90 (-6.69; 1.05)	0.134	Stationary
	Coastline	5.8	-0.93 (-2.27; 0.42)	0.159	Stationary
	Cerrado	6.0	1.96 (0.10; 3.85)	0.040	Increasing

APC: Annual Percent Change; 95%CI: 95% confidence interval.

Source: Hospital Information System of the Brazilian Unified Health System (SIH/SUS/DATASUS); prepared by the authors.

**Figure 2:**
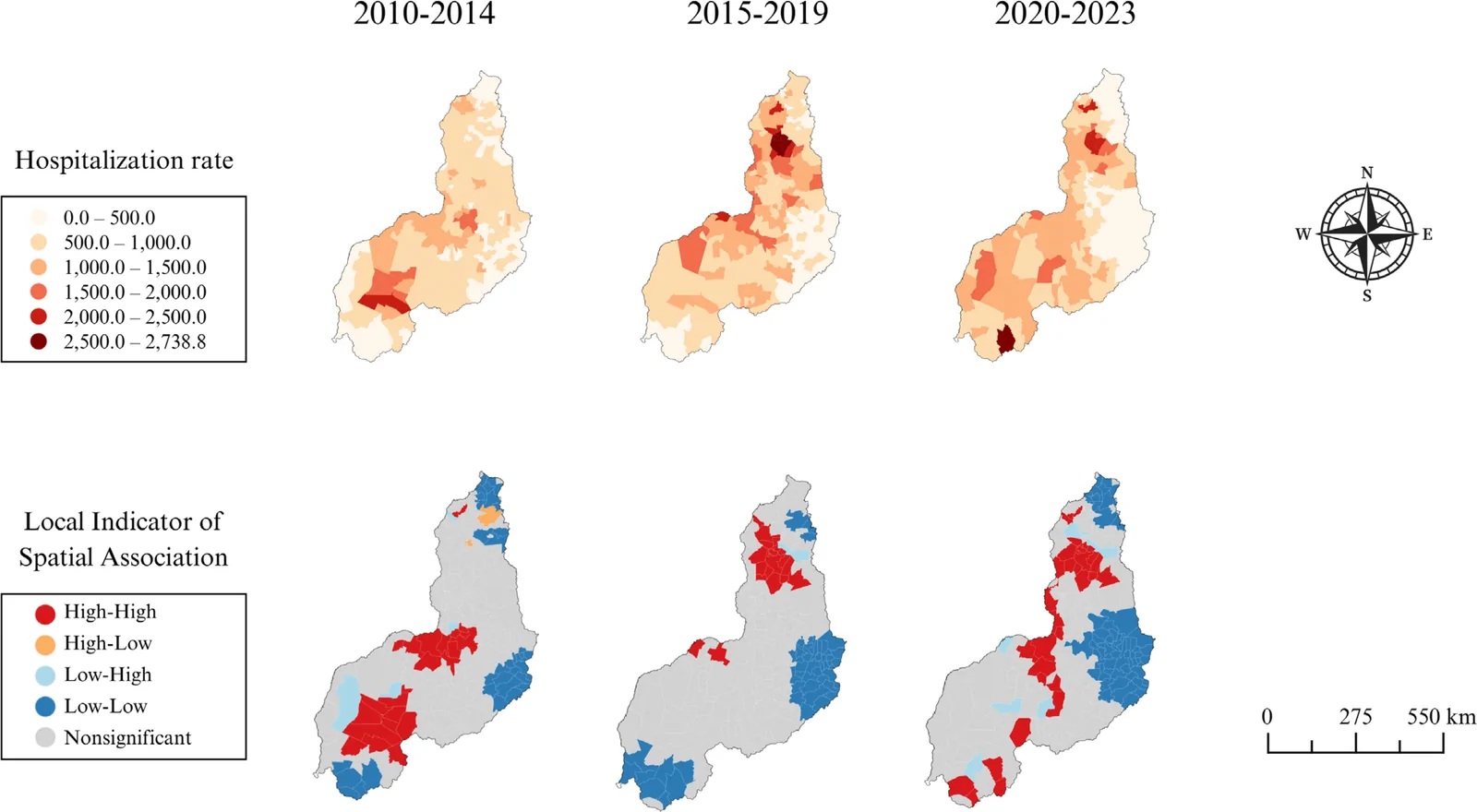
Accumulated rates of hospitalization due to road traffic injuries per 100 thousand inhabitants and Local Indicator of Spatial Association clusters, per municipality, Piauí, 2010–2023 (n=80,251).

Source: Hospital Information System of the Brazilian Unified Health System (SIH/SUS/DATASUS), population projections of the Ministry of Health, and digital territorial meshes of the Brazilian Institute of Geography and Statistics (IBGE); prepared by the authors.

As per the spatial analysis, we identified changes in the geographic distribution of hospitalizations over time. In the 2010–2014 five-year period, the highest rates were concentrated in municipalities in the South of the state, with high-high clusters in this region. In the 2015–2019 period, clusters were identified in the central areas and in part of the North. In the 2020–2023 period, new areas with high rates emerged in the North and South extremes, while low-low clusters persisted in the Southeast of the state (Figure 2).

## DISCUSSION

According to the main findings of this study, there was an increasing trend in the rate of hospitalization due to RTI in Piauí from 2010 to 2023, with a predominance of men, young adults, and motorcyclists, concentrated in the Mid North macroregion. The mean cost per hospitalization showed a decreasing trend, while hospital case fatality and mean length of hospital stay remained stationary. As per the spatial analysis, we identified high-rate clusters in the North and South of the state, with shift over the period.

The high mean rate of hospitalization and its increasing trend indicate that RTI remain a public health problem in Piauí, despite the policies implemented in Brazil in recent decades: The Brazilian Traffic Code (1997), the Drinking and Driving Law (2008) and its update, the New Zero-Tolerance Drinking and Driving Law (Law 12.760/2012)^15,16^, the Life in Traffic Project (*Projeto Vida no Trânsito*), the National Plan for the Reduction of Death and Injuries in Traffic (*Plano Nacional de Redução de Mortes e Lesões no Trânsito* – PNATRANS), and the Strategic Action Plan to Combat Chronic Noncommunicable Diseases (NCDs). However, there was a particular reduction in rates in 2020, possibly associated with decreased mobility during the COVID-19 pandemic. Although the New Zero-Tolerance Drinking and Driving Law has shown an impact on the reduction of hospitalizations at the national level—with an estimated 440,599 hospitalizations avoided between December 2012 and December 2019^17^—the North and Northeast regions did not show immediate reduction in hospitalization rates after its implementation^17^. This finding suggests that the effectiveness of these policies varies according to the regional conditions of inspection and infrastructure, and that the benefits of road safety measures may have reached its limit in the context of Piauí, as already observed in other Brazilian locations^6^.

Motorcyclists had a hospitalization rate 12 times higher than that of pedestrians, with an increasing trend over the period. This finding is consistent with national and international literature^6,18^. Motorcyclists are classified as "vulnerable road users" because they do not have mechanical protection—body, airbags, seat belts—being directly exposed to impact in the event of a collision^19^. In addition, Piauí presents the second highest proportion of households with motorcycles in Brazil (52.1% in 2024)^7^, and the growth of the circulating fleet, especially in the Northeast region^18^, contributes to the progressive increase in hospitalizations. The increasing trend among cyclists, although with lower absolute numbers, deserves attention and may be related to the increase in the use of bicycles as a means of transport without adequate cycling infrastructure.

The highest hospitalization rates were observed among young adults (20–39 years) and adults (40–59 years), both with increasing trend. This pattern is consistent with global GBD data, according to which men aged 15 to 49 years is the group with highest burden of injuries due to RTI worldwide^2^. The greater exposure of this age group is due both to daily commuting to work activities and to more prevalent risk behaviors such as excessive speed and alcohol consumption before driving^20,21^. A direct consequence is the withdrawal of economically-active people from their work activities, generating, in addition to the direct costs of hospitalization, loss of productivity and impacts on social security and family income^4^.

Male prevalence in hospitalizations (rate 4.4 times higher than women) is explained by multiple factors. Data from the National Survey of Health and the Telephone Surveillance System for Noncommunicable Diseases (VIGITEL) show that driving after alcohol consumption is significantly more common among men^20–22^, who also assume more aggressive behavior in traffic^23^. In addition, young men use motorcycles more for commercial purposes, including work on delivery platforms and application transportation. Authors of a Brazilian study on digital platform delivery workers found a 44.1% prevalence of occupational accidents, reaching 54.6% among the youngest (up to 28 years), with 82.8% of these accidents occurring in traffic^24^. This precariousness of work, with long journeys and accelerated pace, increases the exposure to the risk of RTI.

The total cost of hospitalizations in the period was BRL 90.2 million (BRL 128.0 million corrected by the IGP-M), with a mean cost per hospitalization showing a decreasing trend. This trend, however, should not be interpreted as a real reduction in care costs. The SUS procedures table presents a recognized gap in relation to actual costs, and the values recorded in the SIH/SUS do not include the complementary financing of states and municipalities^25^. Thus, the decreasing trend of the mean cost probably reflects the progressive lag of the SUS table in relation to inflation, not an effective reduction of the resources employed in health care. This finding reinforces the need for a review of reimbursement values and complementary analyses that include indirect costs and tripartite financing.

The mean length of stay of 4.9 days remained stable throughout the period. Greater length of hospital stay among older adults is expected, considering the greater physiological fragility, the presence of comorbidities, and the greater severity of injuries in this age group, factors that prolong recovery and increase the risk of complications^26^. The increasing trend of length of hospital stay among vehicle occupants may be related to the greater severity of accidents involving high-energy collisions on highways.

Hospital case fatality of 2.3% remained stable in the period. This stability can be explained by the counterbalance of two mechanisms: on the one hand, improvements in pre-hospital (expansion of the Mobile Emergency Care Service [*Serviço de Atendimento Móvel de Urgência*] – SAMU) and hospital care over the period may have contributed to the reduction of case fatality; on the other hand, the increase in the number of hospitalizations may include proportionally more serious cases, keeping the indicator stable. In high-income countries, hospital case fatality due to trauma has shown a decreasing trend, associated with the maturation of organized trauma systems^27,28^. The stability observed in Piauí may reflect limitations in the organization of the trauma care network in the state, with unequal access to specialized services between regions. The decreasing trend observed among young and older adults and in the Entre Rios and Mid North regions may indicate localized improvements in hospital care in these areas, which include the capital Teresina.

According to the spatial analysis, we verified a progressive concentration of high hospitalization rates in the North and South of the state, with significant high-high clusters in these regions. This distribution can be explained by multiple factors: presence of federal and state highways with great flow, lower road infrastructure (signaling, lighting, and paving) in rural areas, higher proportion of motorcycles as the main means of transport, and lower access to surveillance and education services in traffic. These intra-regional disparities are consistent with the national standard, according to which regions with lower socioeconomic development have higher rates of RTI^3,29^. The emergence of new clusters in the North of the state in the most recent period (2020–2023) may be related to the growth of the fleet of motorcycles and to the disorderly urban expansion in these areas. Low-low clusters in the Southeast of the state may reflect lower population density and lower road flow.

This study presents limitations that should be considered. The coverage of SIH/SUS, which records approximately 75% of hospital admissions in Brazil, may underestimate the actual number of hospitalizations due to RTI, especially in regions with higher participation of the private sector^30^. The monetary values recorded in the SIH/SUS do not include the complementary financing of states and municipalities, underestimating real costs^25^. The high proportion of records with incomplete information, particularly in the Northeast region^29,30^, may limit the accuracy of the estimates. Lastly, the ecological design does not allow causal inferences at the individual level.

We conclude that, in Piauí, between 2010 and 2023, there was an increasing trend in the rate of hospitalization due to RTI, with a predominance of men, young adults, and motorcyclists, while the mean cost showed a decreasing trend and case fatality and mean length of hospital stay remained stationary. As per the spatial analysis, we identified high-rate clusters in the North and South of the state, suggesting the need for regionalized interventions. These findings can support the planning of specific actions: strengthening surveillance on highways of regions with higher rates, expansion of traffic education programs directed to young motorcyclists, review of road infrastructure in areas of greater risk, and adequacy of the trauma care network to reduce hospital case fatality.

## References

[1] GBD 2023 Disease and Injury and Risk Factor Collaborators (2025). Burden of 375 diseases and injuries, risk-attributable burden of 88 risk factors, and healthy life expectancy in 204 countries and territories, including 660 subnational locations, 1990-2023: a systematic analysis for the Global Burden of Disease Study 2023. Lancet.

[2] Wang K, Li Z (2025). Global, regional, and national burdens of road injuries from 1990 to 2021: findings from the 2021 Global Burden of Disease Study. Injury.

[3] Guimarães RA, Sena KG, Morais OL, Malta DC (2023). Magnitude and factors associated with motor road traffic injuries in Brazil: results from the National Health Survey, 2019. Injury.

[4] Andrade SSCA, Mello-Jorge MHP (2016). Mortality and potential years of life lost by road traffic injuries in Brazil, 2013. Rev Saude Publica.

[5] Aquino EC, Antunes JLF, Morais OL (2020). Mortality by road traffic injuries in Brazil (2000-2016): capital cities versus non-capital cities. Rev Saude Publica.

[6] Conceição GMS, Alencar GP, Latorre MRDO (2021). Time trend in hospitalizations from motor vehicle accidents in the city of São Paulo, Brazil, 2000-2019. Cad Saude Publica.

[7] Instituto Brasileiro de Geografia e Estatística Piauí: Cidades e Estados [Internet].

[8] Bittencourt SA, Camacho LAB, Leal MC (2006). O Sistema de Informação Hospitalar e sua aplicação na saúde coletiva. Cad Saude Publica.

[9] Fundação Getulio Vargas (2024). Instituto Brasileiro de Economia. Índices de preços [Internet].

[10] Banco Central do Brasil Calculadora do cidadão: correção de valores [Internet].

[11] Andrade FR, Antunes JLF (2023). Time and memory in time series analysis. Epidemiol Serv Saude.

[12] Antunes JLF, Cardoso MRA (2015). Uso da análise de séries temporais em estudos epidemiológicos. Epidemiol Serv Saude.

[13] Anselin L (1995). Local Indicators of Spatial Association—LISA. Geogr Anal.

[14] Rey SJ, Anselin L (2007). PySAL: a python library of spatial analytical methods. Rev Reg Stud.

[15] Malta DC, Soares AM, Montenegro MMS, Mascarenhas MDM, Silva MMA, Lima CM (2010). Análise da mortalidade por acidentes de transporte terrestre antes e após a Lei Seca - Brasil, 2007-2009. Epidemiol Serv Saude.

[16] Malta DC, Aquino EC, Veloso GA, Teixeira RA, Cunninghanm M, Cardoso LSM (2023). Mortality by road transport injury in Brazilian municipalities between 2000 and 2018. Public Health.

[17] Souza CRL, Russo LX, Silva EN (2022). Association of the new zero-tolerance drinking and driving law with hospitalization rate due to road traffic injuries in Brazil. Sci Rep.

[18] Corgozinho MM, Montagner MA, Rodrigues MAC (2018). Vulnerabilidade sobre duas rodas: tendência e perfil demográfico da mortalidade decorrente da violência no trânsito motociclístico no Brasil, 2004-2014. Cad Saude Colet.

[19] Vecino-Ortiz AI, Nagarajan M, Elaraby S, Guzman-Tordecilla DN, Paichadze N, Hyder AA (2022). Saving lives through road safety risk factor interventions: global and national estimates. Lancet.

[20] Guimarães RA, Morais OL, Santos TMB, Mandacarú PMP, Machado EL, Caiaffa WT (2023). Impact of the program life in traffic and new zero-tolerance drinking and driving law on the prevalence of driving after alcohol abuse in brazilian capitals: an interrupted time series analysis. PLoS One.

[21] Saes-Silva E, Nardes IARS, Melo MER, Silva MP, Bernadina JD, Magalhães MGS (2026). Alcohol consumption and driving in Brazil: a panel study of 731,683 individuals from 2007 to 2022. An Acad Bras Cienc.

[22] Malta DC, Bernal RTI, Mascarenhas MDM, Silva MMA, Szwarcwald CL, Morais OL (2015). Consumo de bebidas alcoólicas e direção de veículos nas capitais brasileiras e no Distrito Federal, segundo dois inquéritos nacionais de saúde. Rev Bras Epidemiol.

[23] Mandacarú PMP, Rabelo IVM, Silva MAA, Tobias GC, Morais OL (2018). Óbitos e feridos graves por acidentes de trânsito em Goiânia, Brasil – 2013: magnitude e fatores associados. Epidemiol Serv Saude.

[24] Siqueira JS, Werneck GL, Pena PGL, Fernandes RCP (2025). Delivery workers’ subordinate work in delivery platform companies and street accidents: magnitude and associated factors. Cad Saude Publica.

[25] Andrade SSCA, Jorge MHPM (2017). Internações hospitalares por lesões decorrentes de acidente de transporte terrestre no Brasil, 2013: permanência e gastos. Epidemiol Serv Saude.

[26] Koch DA, Hagebusch P, Lefering R, Faul P, Hoffmann R, Schweigkofler U (2023). Changes in injury patterns, injury severity and hospital mortality in motorized vehicle accidents: a retrospective, cross-sectional, multicenter study with 19,225 cases derived from the TraumaRegister DGU®. Eur J Trauma Emerg Surg.

[27] Berecki-Gisolf J, Fernando T, D’Elia A (2023). Trends in mortality outcomes of hospital-admitted injury in Victoria, Australia 2001-2021. Sci Rep.

[28] Nathens AB, Jurkovich GJ, Cummings P, Rivara FP, Maier RV (2000). The effect of organized systems of trauma care on motor vehicle crash mortality. JAMA.

[29] GBD 2016 Brazil Collaborators (2018). Burden of disease in Brazil, 1990-2016: a systematic subnational analysis for the Global Burden of Disease Study 2016. Lancet.

[30] Coelho R, Rocha R, Hone T (2024). Improvements in data completeness in health information systems reveal racial inequalities: longitudinal national data from hospital admissions in Brazil 2010-2022. Int J Equity Health.

