# Supplementary material for: Spatiotemporal trends in hospital morbidity indicators due to road traffic injuries in the state of Piauí, Brazil, 2010–2023
Source: Rev Bras Epidemiol. 2026 Aug 24;29:e260044. doi: 10.1590/1980-549720260044 (PMC13506215; doi:10.1590/1980-549720260044)
Supplement: Translation

# Translation: Tendências espaço-temporais dos indicadores de morbidade hospitalar por acidentes de transporte terrestre no Piauí, Brasil, 2010-2023

**DOI:** 10.1590/1980-549720260044.2

**Authors:** Francisco Gabriel Thomaz Bastos, Gabriel Stumpf Bastos Amorim, Francisco Antônio da Cruz Dos Santos, Malvina Thaís Pacheco Rodrigues, Márcio Dênis Medeiros Mascarenhas

**Affiliations:** I Universidade Federal do Piauí – Teresina (PI), Brasil.; II Universidade Federal do Piauí, Centro de Inteligência em Agravos Tropicais Emergentes e Negligenciados – Teresina (PI), Brasil.

**Keywords:** Acidentes de trânsito, Hospitalização, Sistemas de informação hospitalar, Mortalidade, Estudos de séries temporais

## Abstract

**Objetivo:**

Analisar as tendências espaço-temporais dos indicadores de morbidade hospitalar por acidentes de transporte terrestre no Piauí de 2010 a 2023.

**Métodos:**

Estudo ecológico de série temporal e análise espacial com dados do Sistema de Informações Hospitalares do Sistema Único de Saúde. A tendência das taxas de internação segundo regiões de saúde e características sociodemográficas foi analisada por meio da Variação Percentual Anual (VPA) e os Intervalos de Confiança de 95% (IC95%), ambos estimados por regressão de Prais-Winsten. A identificação de aglomerados espaciais foi realizada por meio de mapas LISA.

**Resultados:**

Foram registradas 80.251 internações por acidentes de transporte terrestre no período. A taxa média de internação hospitalar foi de 176,1/100 mil habitantes, com tendência crescente (VPA 5,72; IC95% 1,04; 10,61), enquanto a letalidade (2,3%) manteve-se estável (VPA -2,42; IC95% -5,07; 0,30). O valor médio das internações (R$ 1.123,92) apresentou tendência decrescente (VPA -3,17; IC95% -5,57; -0,71), enquanto a permanência hospitalar média (4,9 dias) manteve-se estável (VPA -1,07; IC95% -2,96; 0,86). Observou-se formação de aglomerados espaciais significativos de altas taxas (*clusters* alto-alto) de internação entre residentes de municípios das regiões Norte e Sul do estado.

**Conclusão:**

Houve aumento da taxa de internação por acidentes de transporte terrestre no Piauí, com redução do valor médio e estabilidade da letalidade e da permanência hospitalar. A maior morbidade concentrou-se nas regiões Norte e Sul, sugerindo interiorização da morbidade hospitalar por lesões de trânsito.

## INTRODUÇÃO

Os Acidentes de Transporte Terrestre (ATT) representam um importante problema de saúde pública global. Segundo o *Global Burden of Disease* (GBD) 2023, as lesões por transporte rodoviário permanecem entre as principais causas de anos de vida ajustados por incapacidade (DALYs) mundialmente, com redução não significativa de 13,3% nas taxas padronizadas por idade entre 2010–2023^1^. Dados do GBD 2021 mostram que, em 2021, ocorreram aproximadamente 103,2 milhões de casos de lesões por transporte rodoviário globalmente, com taxas de mortalidade mais elevadas em países de baixa e média renda (22,6 por 100.000 habitantes), quando comparadas aos países de alta renda². Homens de 15 a 49 anos representam mais de 60% dos anos vividos com incapacidade globalmente^2^.

No Brasil, os ATT apresentam magnitude expressiva. A prevalência de lesões autorrelatadas em 12 meses foi de 2,4% em 2019, com disparidades regionais importantes^3^. A mortalidade atingiu 21,0 óbitos por 100.000 habitantes em 2013, com mais de um milhão de anos potenciais de vida perdidos naquele ano, especialmente na faixa etária de 20–29 anos^4^. Estimativas ajustadas para 2019 sugerem declínio de 25% das mortes desde 2012, embora as estatísticas oficiais subestimem em 31% o número real de óbitos^5^. Motociclistas representam o grupo mais afetado: as mortes aumentaram de 4,1% do total em 1996 para 28,5% em 2013, com aumento de 820% na taxa de mortalidade^4^. Em São Paulo, as taxas de hospitalização de motociclistas voltaram a aumentar a partir de 2015, excedendo 140 por 100.000 habitantes em homens de 20 a 59 anos em 2019^6^.

Entre as políticas públicas implementadas, a Nova Lei Seca (Lei 12.760/2012), que estabeleceu tolerância zero para alcoolemia e punições mais severas, demonstrou impacto significativo na redução da mortalidade e das hospitalizações por ATT^5^. O custo econômico total dos ATT em regiões urbanas foi estimado em aproximadamente US$ 9,9 bilhões em 2006, equivalente a 1,2% do PIB brasileiro daquele ano^4^.

No Piauí, os ATT impõem-se como grande desafio sanitário e social. O estado registrou uma das maiores taxas de crescimento no risco de morrer por ATT no Brasil entre municípios não capitais, com aumento médio de 7,50% ao ano no período de 2000 a 2016^5^.

A análise da morbidade hospitalar segundo regiões de saúde e características sociodemográficas é fundamental para embasar políticas públicas regionais de prevenção e controle. O conhecimento dos padrões espaço-temporais de hospitalização pode orientar a alocação de recursos, o planejamento de serviços de emergência e trauma e a implementação de intervenções preventivas direcionadas às populações e às regiões de maior risco. Adicionalmente, a análise de tendências de custos hospitalares permite avaliar o impacto econômico sobre o sistema de saúde estadual e subsidiar decisões sobre investimentos em prevenção em comparação aos custos do tratamento. O presente estudo teve como objetivo analisar as tendências espaço-temporais dos indicadores de morbidade hospitalar por acidentes de transporte terrestre no Piauí, Brasil, de 2010 a 2023.

## MÉTODOS

### Desenho e período do estudo

Trata-se de estudo ecológico de séries temporais com análise espacial, conduzido com dados sobre internações por ATT envolvendo residentes do Piauí no período de 2010 a 2023. O recorte temporal foi definido considerando-se que 2010 marca o início da Década de Ação pela Segurança no Trânsito, proposta pela Organização das Nações Unidas (ONU), e que 2023 corresponde ao último ano com dados consolidados no sistema de informação utilizado no momento da realização do estudo.

### População do estudo

A população do estudo compreende todas as internações hospitalares por ATT (CID-10: V01-V89) registradas no Sistema de Informações Hospitalares do SUS (SIH/SUS), de residentes do estado do Piauí, no período de 2010 a 2023.

### Local do estudo

O local de estudo é o Piauí, no Nordeste do Brasil. Possui 224 municípios, 12 regiões de saúde e 4 macrorregiões de saúde. A população é de 3.271.199 habitantes, no ano de 2022, e a área territorial é de 251.755,48 km^2^, com densidade populacional de 12,99 habitantes/km^2^. Seu Índice de Desenvolvimento Humano (IDH) foi de 0,690 no ano de 2021, ocupando a 23^a^ posição entre as 27 Unidades da Federação (UFs)^7^. O estado apresenta a segunda maior proporção de motocicletas por domicílio do Brasil. Segundo a Pesquisa Nacional por Amostra de Domicílios Contínua do IBGE, cerca de 597 mil domicílios possuíam motocicleta em 2024, correspondendo a 52,1% dos domicílios do estado^7^.

### Fonte de dados

Os dados sobre morbidade hospitalar foram obtidos do SIH/SUS, sendo trabalhados na forma de dados agregados via TABNET. O SIH/SUS é um sistema de informação de abrangência nacional, alimentado pelas Autorizações de Internação Hospitalar (AIH), que são preenchidas pelos estabelecimentos de saúde que prestam atendimento hospitalar ao SUS. A AIH contém informações sobre diagnósticos, procedimentos realizados, dados demográficos do paciente, tempo de permanência, desfecho da internação e valores pagos. Após o preenchimento, as AIH são processadas pelos gestores municipais e estaduais e consolidadas pelo Departamento de Informática do SUS (DATASUS). O SIH/SUS cobre aproximadamente 75% das internações hospitalares no Brasil, com variações regionais^8^.

Os dados populacionais foram coletados no estudo "Projeção da População das Unidades da Federação por sexo e grupos de idade: 2000–2030", elaborado pelo Ministério da Saúde.

Todos os dados utilizados neste estudo encontram-se disponíveis no *site* eletrônico do DATASUS (https://datasus.saude.gov.br/informacoes-de-saude-tabnet/), os quais foram coletados no mês de agosto de 2024.

### Variáveis do estudo

As internações por ATT foram identificadas por meio do diagnóstico secundário registrado na AIH, que corresponde à causa externa da internação, conforme o Capítulo XX da Classificação Estatística Internacional de Doenças e Problemas Relacionados à Saúde, 10^a^ revisão (CID-10). O diagnóstico secundário, nesse contexto, refere-se à circunstância do acidente que motivou a lesão registrada como diagnóstico principal. As categorias utilizadas foram: pedestre (V01-V09), ciclista (V10-V19), motociclista (V20-V39), ocupante de veículos como automóveis, caminhões, ônibus (V40-V79), outros tipos de transporte ou condição da vítima não especificada (V80-V89) e total de acidentes de transporte terrestre (V01-V89).

Os registros foram estratificados segundo: faixa etária — crianças (0 a 9 anos), adolescentes (10 a 19 anos), adultos jovens (20 a 39 anos), adultos (40 a 59 anos) e idosos (60 anos ou mais); sexo — masculino e feminino; tipo de vítima — pedestre, ciclista, motociclista, ocupante de veículo, outros; macrorregião de saúde de residência e suas respectivas regiões de saúde — Semiárido (composta pelas regiões de saúde Vale do Canindé, Vale do Rio Guaribas, Serra da Capivara e Chapada das Mangabeiras), Meio-Norte (Cocais, Entre Rios, Vale do Sambito e Carnaubais), Litoral (Planície Litorânea e Chapada Vale do Rio Itaim) e Cerrado (Tabuleiros do Alto Parnaíba e Vale dos Rios Piauí e Itaueiras). O valor médio da internação também foi coletado como variável.

### Indicadores de morbidade hospitalar

Calcularam-se os seguintes indicadores para o estado do Piauí e para cada nível de desagregação (sexo, faixa etária, tipo de vítima, região de saúde e macrorregião de saúde):

- taxa de internação hospitalar por 100 mil habitantes: razão entre o número de internações por ATT e a população residente correspondente ao estrato analisado (população total para o Piauí; população masculina ou feminina para o estrato sexo; população do respectivo grupo etário para o estrato faixa etária; e população residente na respectiva região ou macrorregião de saúde para os estratos geográficos), multiplicada por 100.000;
- letalidade hospitalar por 100: razão entre o número de internações por ATT que evoluíram a óbito no hospital e o número de internações por ATT multiplicado por 100;
- valor médio da internação: total pago pelas internações por ATT dividido pelo número de internações por ATT;
- tempo de permanência média de internação hospitalar (dias): total de dias de internação por ATT dividido pelo número de internações por ATT.

Os dados referentes aos valores totais em reais (R$) das internações foram submetidos à correção monetária por meio da aplicação do Índice Geral de Preços –Mercado^9^, utilizando-se a calculadora do Banco Central do Brasil^10^.

### Análise estatística

Os indicadores de morbidade hospitalar (variável dependente: Y) foram transformados em escala logarítmica (log_10_) para estabilizar a variância e permitir a interpretação dos coeficientes como variação percentual. A análise de tendência temporal foi realizada por meio da regressão linear generalizada de Prais-Winsten, que corrige a autocorrelação serial dos resíduos por meio de uma transformação iterativa baseada na estimativa do coeficiente de autocorrelação de primeira ordem (ρ). A presença de autocorrelação serial foi avaliada pela estatística de Durbin-Watson. A partir dos coeficientes β e dos erros-padrão estimados pelo modelo, calculou-se a Variação Percentual Anual (VPA) e os respectivos intervalos de confiança de 95% (IC95%), conforme fórmula^11^: VPA = (10^β^−1)×100. O teste de hipótese utilizado foi o teste t para o coeficiente β da regressão, com nível de significância quando o valor de p foi menor que 0,05. As tendências foram classificadas como crescentes (valor de p<0,05 e β positivo), decrescentes (valor de p<0,05 e β negativo) ou estacionárias (valor de p≥0,05), conforme critérios previamente descritos na literatura^12^.

A análise espacial foi conduzida em três intervalos de tempo (2010–2014, 2015–2019 e 2020–2023), definidos para captar possíveis mudanças nos padrões espaciais ao longo do período. A análise espacial foi restrita à taxa de internação hospitalar por ser o indicador que melhor reflete a distribuição geográfica da carga de morbidade. Inicialmente, foram elaborados mapas coropléticos com as taxas de internação acumuladas dos respectivos períodos. Em seguida, utilizou-se o Indicador Local de Associação Espacial (LISA) para identificar agrupamentos espaciais, classificando os municípios segundo seus padrões de associação como alto-alto, baixo-baixo, alto-baixo e baixo-alto^13^. Apenas os agrupamentos com significância estatística (valor de p<0,05) foram considerados, sendo os demais classificados como não significativos. A matriz de vizinhança foi definida pelo método "Queen".

Os dados foram tabulados no Microsoft Excel e convertidos em indicadores. A análise de tendência foi executada no programa Stata, versão 16.1 (StataCorp LP, College Station, EUA). As análises espaciais foram realizadas no *software* Python, versão 3.10.3, com a biblioteca ESDA^14^, versão 2.7.0, e mapas digitais oficiais do Instituto Brasileiro de Geografia e Estatística (IBGE).

### Aspectos éticos

Este estudo utilizou exclusivamente dados secundários, anonimizados e de acesso público, sem qualquer identificação dos participantes. Portanto, está dispensado de apreciação pelo Comitê de Ética em Pesquisa, conforme as Resoluções do Conselho Nacional de Saúde n^o^ 466/2012 e n^o^ 510/2016, que dispõem sobre a dispensa de avaliação ética para pesquisas com dados públicos.

#### Declaração de disponibilidade de dados:

Todo o conjunto de dados que dá suporte aos resultados deste estudo está disponível mediante solicitação ao autor correspondente. O conjunto de dados não está publicamente disponível devido à integração de bancos de dados realizada pelos autores.

## RESULTADOS

Entre 2010 e 2023, foram registradas 80.251 internações por ATT no Piauí, com taxa média de 176,1 internações/100 mil habitantes e tendência crescente (VPA 5,72; IC95% 1,04 a 10,61). O ano com maior número de internações foi 2016 (n=7.478; 230,3/100 mil hab.) e o menor foi 2012 (n=2.950; 91,8/100 mil hab.), conforme indicado na Tabela 1 e na Figura 1.

**Tabela 1:** Taxa de internação hospitalar por acidentes de transporte terrestre por 100 mil habitantes e tendência temporal, Piauí, 2010–2023 (n=80.251).

Variáveis		Taxa de internação hospitalar	VPA (IC95%)	Valor p	Tendência
Piauí		176,1	5,72 (1,04; 10,61)	0,020	Crescente
Sexo					
	Masculino	292,6	5,71 (0,92; 10,71)	0,023	Crescente
	Feminino	65,9	6,13 (1,77; 10,69)	0,009	Crescente
Idade (em anos)					
	0–9	24,5	5,20 (-2,66; 13,70)	0,180	Estável
	10–19	124,8	4,56 (-0,79; 10,19)	0,089	Estável
	20–39	279,1	4,30 (0,03; 8,74)	0,048	Crescente
	40–59	200,6	7,54 (2,75; 12,55)	0,005	Crescente
	60 ou mais	112,8	8,47 (2,40; 14,90)	0,010	Crescente
Tipo de vítima					
	Pedestre	12,6	-0,60 (-11,50; 11,64)	0,912	Estável
	Ciclista	4,3	13,73 (2,05; 26,74)	0,024	Crescente
	Motociclista	151,2	5,76 (1,32; 10,39)	0,015	Crescente
	Ocupante de veículo	4,7	11,07 (-0,64; 24,16)	0,063	Estável
	Outros	3,3	6,60 (-3,53; 17,8)	0,188	Estável
Região de saúde					
	Carnaubais	272,6	12,92 (1,05; 26,19)	0,035	Crescente
	Chapada das Mangabeiras	171	3,50 (-2,96; 10,39)	0,267	Estável
	Cocais	110,1	3,41 (-2,94; 10,17)	0,271	Estável
	Entre Rios	230,7	8,52 (2,13; 15,31)	0,012	Crescente
	Planície Litorânea	84,4	-1,34 (-24,14; 28,32)	0,913	Estável
	Serra da Capivara	181,9	7,44 (5,65; 9,26)	<0,001	Crescente
	Tabuleiros do Alto Parnaíba	243,1	10,63 (4,04; 17,62)	0,004	Crescente
	Vale do Canindé	171,2	-2,64 (-6,62; 1,52)	0,189	Estável
	Vale do Rio Guaribas	83,8	-13,84 (-19,29; -8,03)	<0,001	Decrescente
	Vale do Sambito	122	-1,29 (-10,03; 8,29)	0,765	Estável
	Vale dos Rios Piauí e Itaueiras	227,2	7,17 (4,24; 10,17)	0,001	Crescente
	Chapada Vale do Rio Itaim	54,2	-11,38 (-20,04; -1,78)	0,025	Decrescente
Macrorregião					
	Semiárido	100,1	-7,20 (-11,62; -2,56)	0,006	Decrescente
	Meio-Norte	235,6	9,03 (2,05; 16,49)	0,015	Crescente
	Litoral	99,5	3,92 (-4,66; 13,27)	0,350	Estável
	Cerrado	199,5	6,23 (4,72; 7,76)	<0,001	Crescente

**VPA:** Variação Percentual Anual; IC95%: Intervalo de Confiança de 95%. Para idade, sexo, região de saúde e macrorregião as taxas foram calculadas utilizando como denominador a população correspondente a cada estrato analisado.

**Fonte:** Sistema de Informações Hospitalares do Sistema Único de Saúde (SIH/SUS/DATASUS) e projeções populacionais do Ministério da Saúde; elaboração dos autores.

**Figura 1:** Série temporal da taxa de internação hospitalar por acidentes de transporte terrestre por 100 mil habitantes, Piauí, Brasil, 2010–2023 (n=80.251).

**Fonte:** Sistema de Informações Hospitalares do Sistema Único de Saúde (SIH/SUS/DATASUS) e projeções populacionais do Ministério da Saúde; elaboração dos autores.

A taxa de internação entre homens (292,6/100 mil homens) foi 4,4 vezes superior à das mulheres (65,9/100 mil mulheres), com tendência crescente em ambos os sexos. Por faixa etária, adultos jovens (20–39 anos) apresentaram a maior taxa média (279,1/100 mil hab.) da faixa etária, seguidos por adultos da faixa etária de 40–59 anos (200,6/100 mil hab.). Ambos os grupos, assim como o de idosos, apresentaram tendência crescente, enquanto crianças e adolescentes apresentaram valores estacionários (Tabela 1).

Motociclistas concentraram a maior carga de internações, com taxa média de 151,2/100 mil habitantes — 12 vezes superior à de pedestres (12,6/100 mil hab.). Apenas motociclistas e ciclistas apresentaram tendência crescente. Entre as regiões de saúde, Carnaubais registrou a maior taxa (272,6/100 mil hab.) da região, e Chapada Vale do Rio Itaim, a menor (54,2/100 mil hab.). Tendência decrescente foi observada em Vale do Rio Guaribas, Chapada Vale do Rio Itaim e na macrorregião Semiárido; as demais apresentaram tendência crescente ou estacionária (Tabela 1).

No período, ocorreram 1.866 óbitos hospitalares, com letalidade média de 2,3% e tendência estacionária. Tendência decrescente da letalidade foi observada apenas na região Entre Rios, na macrorregião Meio-Norte e nas faixas de adultos jovens e idosos. Os demais estratos geográficos apresentaram tendência estacionária (Tabela 2).

**Tabela 2:** Letalidade hospitalar por acidentes de transporte terrestre e tendência temporal, Piauí, 2010–2023 (n=80.251).

Variáveis		Letalidade hospitalar (%)	VPA (IC95%)	Valor p	Tendência
Piauí		2,3	-2,42 (-5,07; 0,30)	0,076	Estável
Sexo					
	Masculino	2,5	-2,38 (-5,15; 0,48)	0,094	Estável
	Feminino	1,6	-2,11 (-4,60; 0,44)	0,096	Estável
Idade (em anos)					
	0–9	1,4	-0,65 (-7,76; 7,02)	0,853	Estável
	10–19	1,7	-0,93 (-5,52; 3,88)	0,675	Estável
	20–39	2,1	-2,99 (-5,58; -0,33)	0,031	Decrescente
	40–59	2,3	-2,55 (-2,55; 0,60)	0,103	Estável
	60 ou mais	5,1	-5,36 (-8,14; -2,50)	0,002	Decrescente
Tipo de vítima					
	Pedestre	2,4	4,33 (-5,96; 15,75)	0,391	Estável
	Ciclista	2,4	1,82 (-8,25; 12,99)	0,713	Estável
	Motociclista	2,3	-2,93 (-6,05; 0,29)	0,071	Estável
	Ocupante de veículo	3,6	4,40 (-5,89; 15,82)	0,384	Estável
	Outros	2,1	-5,69 (-16,93; 7,06)	0,334	Estável
Região de saúde					
	Carnaubais	2,1	-5,95 (-12,19; 0,72)	0,075	Estável
	Chapada das Mangabeiras	1,7	1,41 (-6,73; 10,25)	0,722	Estável
	Cocais	3,6	1,07 (-2,82; 5,12)	0,566	Estável
	Entre Rios	2,1	-5,34 (-8,75; -1,81)	0,007	Decrescente
	Planície Litorânea	1,3	-10,5 (-20,98; 1,38)	0,076	Estável
	Serra da Capivara	2,5	10,42 (0,02; 21,91)	0,050	Estável
	Tabuleiros do Alto Parnaíba	1,7	-1,49 (-8,19; 5,70)	0,651	Estável
	Vale do Canindé	1,9	1,25 (-7,87; 11,28)	0,779	Estável
	Vale do Rio Guaribas	5,1	9,04 (-3,19; 22,81)	0,139	Estável
	Vale do Sambito	3,6	-1,05 (-6,43; 4,65)	0,689	Estável
	Vale dos Rios Piauí e Itaueiras	1,8	0,86 (-3,94; 5,91)	0,709	Estável
	Chapada Vale do Rio Itaim	4,8	-1,35 (-15,08; 14,59)	0,846	Estável
Macrorregião					
	Semiárido	3,7	2,86 (-7,84; 14,80)	0,586	Estável
	Meio-Norte	2,1	-5,21 (-8,92; -1,36)	0,013	Decrescente
	Litoral	2,8	-0,03 (-4,05; 4,16)	0,988	Estável
	Cerrado	1,9	3,17 (-0,53; 7,01)	0,087	Estável

**VPA:** Variação Percentual Anual; IC95%: Intervalo de Confiança de 95%.

**Fonte:** Sistema de Informações Hospitalares do Sistema Único de Saúde (SIH/SUS/DATASUS); elaboração dos autores.

O valor total das internações foi de R$ 90,2 milhões (R$ 128,0 milhões em valores corrigidos pelo IGP-M). O valor médio por internação foi de R$ 1.123,92 (corrigido: R$ 1.659,40), com tendência decrescente (VPA -3,17; IC95% -5,57 a -0,71). Nenhum estrato apresentou tendência crescente para esse indicador (Tabela 3).

**Tabela 3:** Valor médio das internações hospitalares por acidentes de transporte terrestre e tendência temporal, Piauí, 2010–2023 (n=80.251).

Variáveis		Valor médio (R$)	VPA (IC95%)	Valor p	Tendência
Piauí		1.123,92	-3,17 (-5,57; -0,71)	0,016	Decrescente
Sexo					
	Masculino	1.151,85	-3,00 (-5,43; -0,52)	0,022	Decrescente
	Feminino	1.006,70	-3,90 (-6,20; -1,54)	0,004	Decrescente
Idade (em anos)					
	0–9	958,08	-3,44 (-6,47; -0,31)	0,034	Decrescente
	10–19	1.110,28	-4,31 (-6,80; -1,76)	0,003	Decrescente
	20–39	1.126,98	-2,82 (-5,28; -0,29)	0,032	Decrescente
	40–59	1.118,48	-3,23 (-5,38; -1,03)	0,008	Decrescente
	60 ou mais	1.187,77	-3,22 (-6,09; -0,26)	0,035	Decrescente
Tipo de vítima					
	Pedestre	974,28	-4,53 (-14,18; 6,21)	0,362	Estável
	Ciclista	984,04	0,80 (-1,02; 2,64)	0,360	Estável
	Motociclista	1.132,64	-2,96 (-5,14; -0,73)	0,014	Decrescente
	Ocupante de veículo	1.258,30	2,51 (-1,50; 6,68)	0,201	Estável
	Outros	1.283,86	-8,28 (-15,15; -0,85)	0,032	Decrescente
Macrorregião					
	Semiárido	1.191,10	-1,29 (-5,60; 3,21)	0,537	Estável
	Meio-Norte	1.155,13	-3,69 (-6,89; -0,38)	0,032	Decrescente
	Litoral	1.124,11	-7,03 (-11,80; -2,00)	0,011	Decrescente
	Cerrado	1.005,40	-2,15 (-3,66; -0,62)	0,010	Decrescente

**VPA:** Variação Percentual Anual; IC95%: Intervalo de Confiança de 95%.

**Fonte:** Sistema de Informações Hospitalares do Sistema Único de Saúde (SIH/SUS/DATASUS); elaboração dos autores.

As internações totalizaram 392.047 dias de permanência, com tempo médio de 4,9 dias e tendência estacionária. A permanência média foi predominantemente estável, com tendência decrescente apenas entre crianças e adolescentes e crescente entre ocupantes de veículos (Tabela 4).

**Tabela 4:** Permanência média (dias) das internações hospitalares por acidentes de transporte terrestre e tendência temporal, Piauí, 2010–2023 (n=80.251).

Variáveis		Permanência média (dias)	VPA (IC95%)	Valor p	Tendência
Piauí		4,9	-1,07 (-2,96; 0,86)	0,248	Estável
Sexo					
	Masculino	4,9	-1,00 (-2,92; 0,96)	0,287	Estável
	Feminino	4,6	-1,38 (-3,21; 0,48)	0,130	Estável
Idade (em anos)					
	0–9	3,6	-2,26 (-3,29; -1,22)	0,001	Decrescente
	10–19	4,7	-1,57 (-2,99; -0,13)	0,035	Decrescente
	20–39	4,8	-1,22 (-3,04; 0,62)	0,173	Estável
	40–59	5,1	-1,36 (-3,50; 0,82)	0,198	Estável
	60 ou mais	5,5	-1,12 (-3,19; 0,98)	0,266	Estável
Tipo de vítima					
	Pedestre	5,4	-0,9 (-2,05; 0,27)	0,119	Estável
	Ciclista	4,2	-1,05 (-3,96; 1,93)	0,453	Estável
	Motociclista	4,8	-1,12 (-3,29; 1,10)	0,290	Estável
	Ocupante de veículo	5,7	5,84 (1,15; 10,74)	0,018	Crescente
	Outros	6,3	-2,9 (-5,74; 0,02)	0,051	Estável
Macrorregião					
	Semiárido	5,3	1,42 (0,06; 2,81)	0,042	Crescente
	Meio-Norte	4,2	-2,90 (-6,69; 1,05)	0,134	Estável
	Litoral	5,8	-0,93 (-2,27; 0,42)	0,159	Estável
	Cerrado	6,0	1,96 (0,10; 3,85)	0,040	Crescente

**VPA:** Variação Percentual Anual; IC95%: Intervalo de Confiança de 95%.

**Fonte:** Sistema de Informações Hospitalares do Sistema Único de Saúde (SIH/SUS/DATASUS); elaboração dos autores.

A análise espacial identificou mudanças na distribuição geográfica das internações ao longo do tempo. No quinquênio 2010–2014, as maiores taxas concentraram-se em municípios do Sul do estado, com *clusters* alto-alto nessa região. No período 2015–2019, os aglomerados foram identificados nas áreas centrais e em parte do Norte. No período 2020–2023, novas áreas com taxas elevadas emergiram nos extremos Norte e Sul, enquanto *clusters* baixo-baixo persistiram no Sudeste do estado (Figura 2).

**Figura 2:** Taxas acumuladas de internação hospitalar por acidentes de transporte terrestre por 100 mil habitantes e *clusters Local Indicator of Spatial Association*, por município, Piauí, 2010–2023 (n=80.251).

**Fonte:** Sistema de Informações Hospitalares do Sistema Único de Saúde (SIH/SUS/DATASUS), projeções populacionais do Ministério da Saúde e malhas territoriais digitais do Instituto Brasileiro de Geografia e Estatística (IBGE); elaboração dos autores.

## DISCUSSÃO

Os principais achados deste estudo revelaram tendência crescente da taxa de internação hospitalar por ATT no Piauí de 2010 a 2023, com predomínio de vítimas do sexo masculino, adultos jovens e motociclistas, concentradas na macrorregião Meio-Norte. O valor médio por internação apresentou tendência decrescente, enquanto a letalidade hospitalar e a permanência média mantiveram-se estáveis. A análise espacial identificou *clusters* de taxas elevadas no Norte e no Sul do estado, com deslocamento ao longo do período.

A elevada taxa média de internação hospitalar e sua tendência crescente indicam que os ATT permanecem como problema de saúde pública no Piauí, a despeito das políticas implementadas no Brasil nas últimas décadas: o Código de Trânsito Brasileiro (1997), a Lei Seca (2008) e sua atualização com tolerância zero (Lei 12.760/2012)^15,16^, o Projeto Vida no Trânsito, o Plano Nacional de Redução de Mortes e Lesões no Trânsito (PNATRANS) e o Plano de Ações Estratégicas para o Enfrentamento das Doenças Crônicas e Agravos Não Transmissíveis (DANT). Observa-se, contudo, redução pontual das taxas em 2020, possivelmente associada à diminuição da mobilidade durante a pandemia de COVID-19. Embora a Nova Lei Seca tenha demonstrado impacto na redução de hospitalizações em nível nacional — com estimativa de 440.599 hospitalizações evitadas entre dezembro de 2012 e dezembro de 2019^17^ —, as regiões Norte e Nordeste não apresentaram redução imediata nas taxas de internação após sua implementação^17^. Esse achado sugere que a efetividade dessas políticas varia conforme as condições regionais de fiscalização e infraestrutura, e que os benefícios das medidas de segurança viária podem ter atingido seu limite no contexto piauiense, conforme já observado em outras localidades brasileiras^6^.

Os motociclistas apresentaram taxa de internação 12 vezes superior à dos pedestres, com tendência crescente ao longo do período. Esse achado é consistente com a literatura nacional e internacional^6,18^. Motociclistas são classificados como "usuários vulneráveis do sistema viário" por não disporem de proteção mecânica — carroceria, *airbags*, cintos de segurança —, ficando diretamente expostos ao impacto em caso de colisão^19^. Além disso, o Piauí apresenta a segunda maior proporção de domicílios com motocicleta do Brasil (52,1% em 2024)^7^, e o crescimento da frota circulante, especialmente na região Nordeste^18^, contribui para o aumento progressivo das internações. A tendência crescente entre ciclistas, embora com números absolutos menores, merece atenção e pode estar relacionada ao aumento do uso da bicicleta como meio de transporte sem infraestrutura cicloviária adequada.

As maiores taxas de internação foram observadas entre adultos jovens (20–39 anos) e adultos (40–59 anos), ambos com tendência crescente. Esse padrão é consistente com dados globais do GBD, que identificam homens de 15 a 49 anos como o grupo de maior carga de lesões por ATT mundialmente^2^. A maior exposição dessa faixa etária decorre tanto do deslocamento diário para atividades laborais quanto de comportamentos de risco mais prevalentes, como velocidade excessiva e consumo de álcool antes de dirigir^20,21^. Uma consequência direta é o afastamento de pessoas economicamente ativas de suas atividades laborais, gerando, além dos custos diretos da internação, perda de produtividade e impactos sobre a previdência e a renda familiar^4^.

A predominância masculina nas internações (taxa 4,4 vezes superior à feminina) é explicada por múltiplos fatores. Dados da Pesquisa Nacional de Saúde e do VIGITEL mostram que dirigir após consumo de álcool é significativamente mais comum entre homens^20–22^, que também assumem comportamentos mais agressivos no trânsito^23^. Adicionalmente, homens jovens utilizam mais as motocicletas para fins comerciais, incluindo trabalho em plataformas de entrega e transporte por aplicativo. Estudo brasileiro com entregadores de plataformas digitais encontrou prevalência de acidentes ocupacionais de 44,1%, chegando a 54,6% entre os mais jovens (até 28 anos), sendo 82,8% desses acidentes ocorridos no trânsito^24^. Essa precarização do trabalho, com jornadas extensas e ritmo acelerado, amplia a exposição ao risco de ATT.

O valor total das internações no período foi de R$ 90,2 milhões (R$ 128,0 milhões corrigidos pelo IGP-M), com valor médio por internação apresentando tendência decrescente. Essa tendência, contudo, não deve ser interpretada como redução real dos custos assistenciais. A tabela de procedimentos do SUS apresenta reconhecida defasagem em relação aos custos reais, e os valores registrados no SIH/SUS não incluem o financiamento complementar de estados e municípios^25^. Assim, a tendência decrescente do valor médio provavelmente reflete a progressiva defasagem da tabela SUS frente à inflação, e não uma redução efetiva dos recursos empregados no atendimento. Esse achado reforça a necessidade de revisão dos valores de reembolso e de análises complementares que incluam custos indiretos e financiamento tripartite.

A permanência média de 4,9 dias manteve-se estável ao longo do período. A maior permanência hospitalar entre idosos é esperada, considerando a maior fragilidade fisiológica, a presença de comorbidades e a maior gravidade das lesões nessa faixa etária, fatores que prolongam a recuperação e aumentam o risco de complicações^26^. A tendência crescente da permanência hospitalar entre ocupantes de veículos pode estar relacionada à maior gravidade dos acidentes envolvendo colisões de alta energia em rodovias.

A letalidade hospitalar de 2,3% manteve-se estável no período. Essa estabilidade pode ser explicada pelo contrabalanço de dois mecanismos: por um lado, melhorias no atendimento pré-hospitalar (expansão do Serviço de Atendimento Móvel de Urgência – SAMU) e hospitalar ao longo do período podem ter contribuído para a redução da letalidade; por outro lado, o aumento do volume de internações pode incluir proporcionalmente mais casos graves, mantendo o indicador estável. Em países de alta renda, a letalidade hospitalar por trauma tem apresentado tendência decrescente, associada à maturação dos sistemas de trauma organizados^27,28^. A estabilidade observada no Piauí pode refletir limitações na organização da rede de atenção ao trauma no estado, com acesso desigual a serviços especializados entre as regiões. A tendência decrescente observada entre adultos jovens e idosos e nas regiões Entre Rios e Meio-Norte pode indicar melhorias localizadas na assistência hospitalar nessas áreas, que incluem a capital Teresina.

A análise espacial revelou concentração progressiva de elevadas taxas de internação no Norte e no Sul do estado, com *clusters* alto-alto significativos nessas regiões. Essa distribuição pode ser explicada por múltiplos fatores: presença de rodovias federais e estaduais de grande fluxo, menor infraestrutura viária (sinalização, iluminação e pavimentação) em áreas rurais, maior proporção de motocicletas como meio de transporte principal e menor acesso a serviços de fiscalização e educação no trânsito. Essas disparidades intrarregionais são consistentes com o padrão nacional, em que regiões com menor desenvolvimento socioeconômico apresentam maiores taxas de lesões por ATT^3,29^. O surgimento de novos *clusters* no Norte do estado no período mais recente (2020–2023) pode estar relacionado ao crescimento da frota de motocicletas e à expansão urbana desordenada nessas áreas. Os *clusters* baixo-baixo no Sudeste do estado podem refletir menor densidade populacional e menor fluxo viário.

Este estudo apresenta limitações que devem ser consideradas. A cobertura do SIH/SUS, que registra aproximadamente 75% das internações hospitalares no Brasil, pode subestimar o número real de internações por ATT, especialmente em regiões com maior participação do setor privado^30^. Os valores monetários registrados no SIH/SUS não incluem o financiamento complementar de estados e municípios, subestimando os custos reais^25^. A elevada proporção de registros com informações incompletas, particularmente na região Nordeste^29,30^, pode limitar a precisão das estimativas. Por fim, o desenho ecológico não permite inferências causais em nível individual.

Conclui-se que, no Piauí, entre 2010 e 2023, houve tendência crescente na taxa de internação hospitalar por ATT, com predomínio de homens, adultos jovens e motociclistas, enquanto o valor médio apresentou tendência decrescente e a letalidade e a permanência hospitalar média mantiveram-se estáveis. A análise espacial identificou *clusters* de taxas elevadas no Norte e no Sul do estado, sugerindo necessidade de intervenções regionalizadas. Esses achados podem subsidiar o planejamento de ações específicas: fortalecimento da fiscalização em rodovias das regiões com maiores taxas, ampliação de programas de educação no trânsito direcionados a motociclistas jovens, revisão da infraestrutura viária nas áreas de maior risco e adequação da rede de atenção ao trauma para reduzir a letalidade hospitalar.
